# Plagiarisms

**Published:** 1874-09-01

**Authors:** 


### PLAGIARISMS.

ONE of the secular journals of our city recently published an editorial notice of the plagiarisms found in the Valedictory Address delivered at the last Annual Commencement of the Rush Medical College of our city. In this article, reference is made to the late exposure in the columns of the EXAMINER, of the plagiarisms discovered in an address incorporated with the transactions of our State Society.

The facts collated by the secular paper alluded to, are facts, indeed. They had come to our knowledge some time before the exposure was made public, and we had verified their truth by actual comparison of the published address with some of the pages, from which entire paragraphs had been taken without acknowledgment by the use of quotation marks or authors' names.

We have delayed calling attention to it mainly through fear that our motives might be misunderstood; but it is the plain duty of the medical journalist to call attention to any and all instances of violation of those rules that should govern writers in relation to the works of others, for in no other way can professional honor and literary fairness be maintained.

The author of the Valedictory Address, like the authors of the addresses before the Illinois and Michigan State Medical Societies, is a personal friend, for whom we have entertained the highest respect. Furthermore, we know all these gentlemen possess abundant ability and command of language, to do justice to any subject without borrowing from others. Hence, we have been all the more surprised and chagrined on seeing their productions sent forth to the world, so largely made up of the unacknowledged language and thoughts of others. Our chagrin has been further deepened by the fact that these gentlemen occupy positions in the profession which should make them scrupulously careful of the example they set. There can be no sectarian feeling in considering a question of this character. We all share in the honor reflected upon us by those who distinguish themselves and our common profession in the world of science or literature, and we do none the less proportionately suffer when one of us is dishonored in the eyes of the public. No one of us can, if he would, release himself from the sensitive and sympathetic bond that unites us. Each of us enforces it by an honorable and useful career. Each of us subjects it to a test, when he is guilty of an unjustifiable act. And of all men, those who hold official and honorable positions, should be the last to allow either indolence or ambition to so far beguile their consciences or obscure their sense of propriety, as to exhibit themselves before the public in borrowed plumage. Detection, sooner or later, is certain. We need indulge in no word of harshness towards the author of the address which has elicited these remarks. Detection and public exposure is sufficient punishment. We would not add to its severity or make it more unendurable. We discharge our duty to the medical public by acknowledging the facts to which we have referred, and we sincerely trust that the example may finally end the abuse of confidence of which he is guilty, who addresses himself, either orally or in print, to his fellow medical men, in thoughts and words that are borrowed without credit from the stores of wisdom accumulated by others with infinite toil.

